# Mapping and quantifying perceptions of environmental change in Kilombero Valley, Tanzania

**DOI:** 10.1007/s13280-019-01226-6

**Published:** 2019-07-25

**Authors:** Emma Li Johansson, Abdulhakim M. Abdi

**Affiliations:** 1 Department of Physical Geography and Ecosystem Science, Lund University, Sölvegatan 12, 223 62 Lund, Sweden; 2 Centre for Environmental and Climate Research, Lund University, Sölvegatan 37, 223 62 Lund, Sweden

**Keywords:** Deforestation, Large-scale land acquisitions, Mixed methods, Participatory research, Remote sensing, Socio-environmental change

## Abstract

Large-scale land acquisitions are a critical driver of global socio-environmental change, in particular in the Global South. This study aims to investigate drivers, impacts and feedbacks of environmental change in Kilombero Valley, Tanzania, a region in rapid societal and environmental transformation. We use land cover classifications to map and quantify land cover changes from 1990 to 2016, and compare these with local perceptions of change from fieldwork conducted in 2015 and 2016. The land change detection clearly shows farmland expansion over the wetlands, which is in line with local perceptions, but fails to detect changes in forest cover, which contradicts local perceptions that proclaim rapid deforestation over the past decade. In order to move towards more inclusive sustainable development, there is a need to break the power asymmetries between agribusinesses and local farmers. Our approach posits that policies of importing regions and countries where land is leased need to be strengthened to make sure that agribusinesses establish farms that are consistent with local and domestic needs. Such policies should not only be informed by assessments of environmental change, but also be guided by local knowledge, needs and future aspirations.

**Electronic supplementary material:**

The online version of this article (10.1007/s13280-019-01226-6) contains supplementary material, which is available to authorized users.

## Introduction

Economic development through foreign agricultural investments is a major driver of land use and land cover (LULC) change, which in turn contributes to critical sustainability challenges due to its undesirable effects on the climate system, water resources, biodiversity and human welfare (Turner et al. 2007; Lambin and Meyfroidt 2011). Land system science aims to understand the dynamics of LULC change as a coupled human–environment system (Turner et al. 2007; Turner and Robbins 2008; Verburg et al. 2015), focusing on the spatio-temporal patterns of change, as well as the underlying socio-economic and environmental drivers, impacts and feedbacks of land system change. A contemporary challenge within land system science is to understand the local effects of increased distal land connections due to the growing competition for land and water resources (Seto and Reenberg 2014).

Over the past two decades, there has been a rapid increase in large-scale land acquisitions to produce fibre, biofuels, feed and food for international markets, which has impacted ecosystems, agro-ecosystems and societies, predominantly across the Global South (D’Odorico et al. 2017). Socio-environmental changes in the context of large-scale land acquisitions are highly complex and associated with several sustainability challenges, like deforestation (Davis et al. 2015), water scarcity and pollution (Dell’Angelo et al. 2018), soil degradation (Lazarus 2014) and food insecurity (Havnevik et al. 2011). The use of natural resources by foreign actors also often leads to conflicts over land and water resources between local and non-local land users (Schoneveld 2014).

Large-scale land acquisitions are rapidly transforming ecosystems and societies in many low-income countries of the world, especially in Sub-Saharan Africa (Messerli et al. 2014; Seaquist et al. 2014). African agriculture is often depicted as stagnant, underproductive and in need of modernisation and intensification (Van Ittersum et al. 2016), and responsible investments in agriculture could have the potential to spur economic development, boost agricultural yields and contribute to food security (Deininger and Byerlee 2011). But critics describe the current trend of land acquisitions as a form of land grab due to unequal power dynamics, involuntary transfer of land rights from small-scale farmers to powerful foreign or domestic investors (Edelman et al. 2013; Davis et al. 2014) and a large focus on producing non-edible crops for export (Johansson et al. 2016).

### Land acquisition and land-use change in Kilombero Valley, Tanzania

One reason for the large amount of land deals in Tanzania (and particularly Kilombero Valley) is the initiative “Southern Agricultural Growth Corridor of Tanzania” (SAGCOT), which was launched in 2011 in order to coordinate agribusiness partnership between the Government of Tanzania, private companies and international donors to improve food security by reducing yield gaps and rural poverty, and also sustain the environment (SAGCOT 2018). It is however questionable to what extent this has been achieved (Bergius et al. 2018).

The area of focus in this study is located in Kilombero Valley and is one of the targeted regions for SAGCOT. It is a region with rapid expansion of foreign and domestic investors, where companies transform natural vegetation or small-scale farming areas to large-scale teak, sugarcane and rice plantations (Table S1). Multiple studies have recently assessed LULC change in Kilombero Valley by using remote sensing, and point to a decline in wetland and forest area due to farmland expansion from year 1990 to 2016 (Leemhuis et al. 2017; Msofe et al. 2019; Munishi-Kongo and Jewitt 2019).

**Fig. 1:**
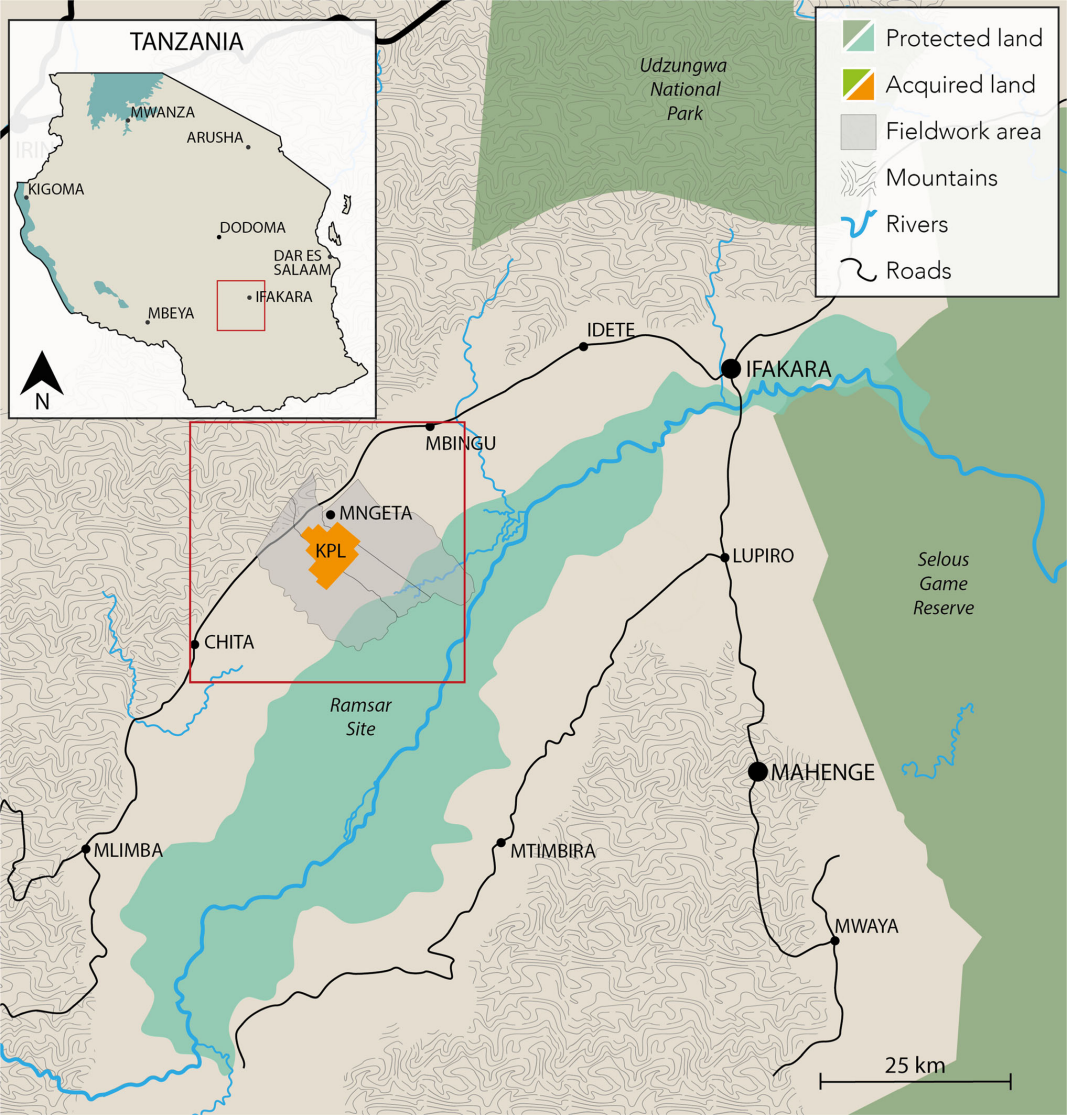
Map of Kilombero Valley, Tanzania, showing the distribution of protected land, acquired land and the village land where fieldwork was conducted

Here we focus on a specific part of the Kilombero Valley in order to compare site-specific local experiences of LULC change, with what can be observed in satellite imagery over the same area. We focus on the area around Kilombero Plantations Limited (KPL), entitled 5800 ha of land in 2007, in order to grow rice. The rice produced at this plantation has not been sold locally, but mainly to larger Tanzanian cities, and neighbouring countries (Land Matrix 2019). The KPL farm covers large parts of three villages (indicated with grey shade in Fig. 1). When KPL arrived in 2007, the company displaced 630 families that lived and farmed the area, in order to drain and clear the land for rice plantations (Johansson, pers. comm.).

#### Climate and ecosystems

Kilombero Valley is called the “Breadbasket of East Africa” due to its ideal conditions for agriculture with its fertile soils, and abundant water (Mombo et al. 2011a, b). The area receives 2000–3100 mm of rainfall per year over two rainy seasons from March to May, and October to December. The uneven rainfall distribution contributes to considerable seasonal variations in water flow, creating a wide variety of wetland types. Multiple rivers feed the floodplain, which covers approximately 8000 km^2^, making it one of the largest freshwater wetlands in East Africa (Kangalawe and Liwenga 2005). The interactions between water, soils, topography, plants and animals make the area a biodiversity hotspot and a highly productive ecosystem (Mombo et al. 2011b). Some important functions of the wetland are groundwater recharge and discharge, flood control, nutrient cycling and a water supply for agriculture, fisheries and industrial use. Its ecological importance, and agricultural potential, has created strong global and local interest to both protect and exploit the area, which currently contains game reserves, national parks (Selous, Udzungwa), conservation areas (Ramsar), as well as multiple large-scale agricultural plantations (Fig. 1).

#### People and livelihoods

The main economic activity in Kilombero District is farming, which employs about 79% of the population, while livestock keepers and fishermen make up less than 1% of the district’s population (NBS 2016). From 2002 to 2012, the rural population increased from 321 611 to 407 880 (NBS 2012), partly driven by an influx of pastoralist and agro-pastoralist groups like Maasai, Sukuma and Barbaig, as well as business people, from all over the country (Nindi et al. 2014). These livelihoods closely connect people to the environment, which make them particularly vulnerable to environmental change. Farmers mainly engage not only in subsistence agriculture, but also forestry and commercial agriculture. Most farms are small, with an average size of 1 ha, but about 10% of the farmers have farmland that exceeds 4 ha (Mombo et al. 2011b). Maize is the main staple crop of the region and dominates agricultural production, but households also grow rice, cassava, banana and other crops.

### Mixed methods for identifying socio-environmental change

Remote sensing and cross-tabulation of classified satellite images are well-established methods for quantifying and tracking LULC change across time and space (Brannstrom and Vadjunec 2014), and has a good record for informing natural resource management (DeFries 2008). If also relating the observed changes to experiences on the ground, remote sensing can be used to identify underlying societal drivers and land-use practices that give rise to environmental change, and how the changes in turn affect people in terms of, e.g. culture and gender (Liverman et al. 1998; Robbins 2003). There is a growing recognition that interdisciplinary research and co-production of knowledge is needed to better understand drivers, impacts and feedbacks of land system change, by linking experiences on the ground to pixels and patterns in satellite images (Liverman et al. 1998; Brannstrom and Vadjunec 2014; Herrmann et al. 2014).

In this article, we combine top-down and bottom-up methods in order to shed light on socio-economic and environmental changes in an area subjected to large-scale land acquisitions. We build on two separate research projects in Kilombero Valley, Tanzania; one which is based on LULC classifications and change detection from remote sensing (Leemhuis et al. 2017); and one based on co-production of knowledge and perceptions of socio-environmental change (Johansson and Isgren 2017). In this way we are able to develop insights of the local meaning and experience of environmental change, while also exploring the spatial extent and remote visibility of change. This mixed methods approach enables us to answer (1) what are the dominant narratives of drivers, impacts and feedbacks of socio-economic and environmental change identified with participatory research approaches? (2) What are the LULC changes observed with remote sensing between 1990, 2004 and 2016? (3) To what extent can perceptions of change be identified in the satellite imagery and land change detection?

## Materials and methods

### Participatory methods

Local narratives of socio-environmental change were documented during fieldwork in Kilombero Valley in March and April, 2015 and 2016. The fieldwork relies on participatory methods to explore natural resource use and local perceptions of socio-environmental change in villages that lease land to foreign agribusinesses (see Johansson and Isgren 2017 for full details).

In total, five focus group discussions were held: three with mixed participants representing different age groups, livelihoods and gender (one in each village where land has been acquired by KPL). Three of the focus group discussions included a mix of farmers, fishermen/farmers and pastoralists/agro-pastoralists, with an equal representation of gender and age groups (approximately 12 people in each focus group, see Fig. S1 for details). These participants were purposefully chosen since they represent the dominant livelihoods in the area, and directly depend on land and water resources. We included a range of younger and older participants in order to get an idea of how past land use compares with the current situation. Questions were open-ended and focussed on (1) natural resource use and change over the last decade, (2) reasons for change and (3) how different livelihood strategies have been affected by the perceived environmental change. We also arranged two additional focus groups with pastoralists/agro-pastoralists, and fishermen. In this way it was possible to also visit new villages that have established in the area due to the expansion of farmland and grazing towards the wetland. The focus group discussions made it possible to outline key landscape features (e.g. forests, rivers, wetland, farmland), and highlighted main experiences and concerns about socio-economic and environmental change, which formed the basis for a participatory painting workshop.

**Fig. 2:**
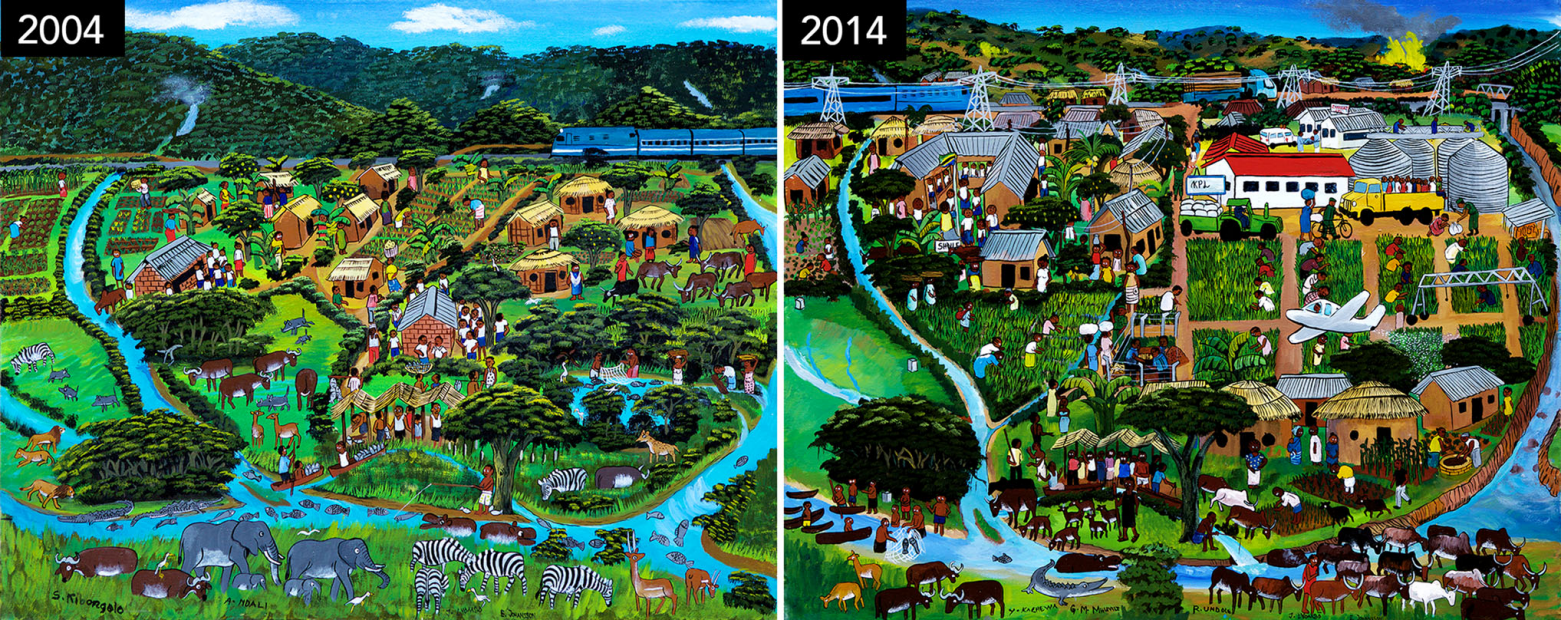
Paintings from participatory art workshop in Kilombero 2015, representing the past and present socio-environmental state (Johansson and Isgren 2017)

Participants for the painting workshop (two for each painting) were selected from one of the focus groups in order to depict the socio-environmental changes of the village and nearby environment (Fig. 2). The paintings were based on narratives that emerged from the focus group discussions, but details could be added, modified and explained by people who passed by and “peer-reviewed” what was visualised. Actually, no by-passer highlighted any errors, but rather added more details to the narratives, or confirmed what they already saw in the image. The focus on different time periods was a means to capture how people experience changes in their environment, including the relation between community and other land users. The purpose of using art as opposed to other participatory methods was to use the paintings for discussing issues of socio-environmental change in an alternative way, both during fieldwork and afterward, in different forums outside of academia, e.g. through exhibitions at the National Museum in Dar Es Salaam.

Several interviews were held along with focus group discussions and the painting workshop. These included key informants like land managers and decision makers at village and district level, company employees (KPL), district-level officials, researchers, civil society organisations and other people (often subsistence farmers) that passed by the village centre where the painting workshop was held. A second field visit was conducted in March 2016 in order to observe changes in the wetland, farmland area and mountain forest. On this occasion, a transect walk in the mountain was done together with a forest ranger (map of transect in Fig. S2).

### Land cover change analysis

The data used for LULC change detection are based on land cover classifications of satellite images produced by Leemhuis et al. (2017). Three time slices, 1990, 2004 and 2016, were selected in order to analyse the temporal development of the study area. These time periods are important for understanding LULC change in the context of large-scale land acquisitions, as they represent the environmental conditions well before KPL was established (1990), the approximate time of company establishment (2004), as well as the current state (2016).

The 1990 and 2004 LULC maps were produced from Landsat 5 Thematic Mapper (TM) using reference data collected from the Collect Earth platform (Bey et al. 2016). The satellite data were standard Level-1TP that had undergone terrain and precision correction to provide radiometric and geodetic accuracy. Normalisation for sensor viewing and solar illumination geometry, atmospheric correction and surface reflectance generation was performed using the Landsat Ecosystem Disturbance Adaptive Processing System (LEDAPS) following the method outlined in Masek (2006).

The 2016 LULC map was created using the Sentinel-2 Multispectral Instrument (MSI), using reference data from two flight campaigns in January and June 2016. The Sentinel-2 data came in a Level-1C processing format that underwent geometric and radiometric correction (Drusch et al. 2012). Correction for the effect of the atmosphere was performed using Sen2Cor (v2.4) to convert the data into Level-2A (Müller-Wilm 2018). In order to harmonise the disparate resolutions, the 30 m Landsat data and the 10 m MSI data were resampled to a common 20-m grid cell size using bilinear interpolation.

LULC classes identified in the study area are presented in Table 1. Classification was performed using a supervised image segmentation-based maximum likelihood classifier in GEOclassifier v1.4.8. Using a statistical learning algorithm such as maximum likelihood in a pixel-based classification of multiple LULC classes using a small amount of training data generally yields low accuracies compared to machine learning methods such as random forests (Khatami et al. 2016). However, the algorithm produces reasonably high accuracies when each class is described properly with ample training data. Here, the training samples were selected from the segmentation outputs based on their spectral similarity. The segmentation was based on (1) a set tolerance that groups pixels into objects based on their spectral characteristics and spatial arrangement, and (2) a minimum surface mapping area that regulates the minimum segment size. The tolerance was set to 30 and, considering the 20 m spatial resolution, the minimum area was set to 3600 m^2^.

**Table 1:** Land use and land cover classes and their description

Class	Description
Wetland	Land with a permanent mixture of water and herbaceous or woody vegetation
Forest	Land with at least 1 hectare in size of which at least 25% is covered by tree canopy with a minimum height of 3 m
Rainfed farmland	Land actively used to grow agriculture crops, including agroforestry systems, wooded crops, herbaceous crops and grain crops
Water	Oceans, seas, lakes, reservoirs and rivers
Irrigated farmland	Crops irrigated permanently or periodically, using permanent infrastructure (irrigation channels, drainage network)
Settlement	Land that includes a settlement

Validation of the classification was performed for each time slice using 400 randomly distributed samples. These were collected using CollectEarth (Bey et al. 2016), a participatory platform developed by the Food and Agriculture Organization for structured data collection based on visual interpretation of high-resolution satellite imagery. Error-adjusted area estimations were calculated following the good practices guide by Olofsson et al. (2014). Overall accuracies of the classification produced by Leemhuis et al. (2017) for the entire Kilombero Valley are above 80% for the 1990 image, and above 90% for 2004 and 2016 (Tables S2, S3).

LULC change detection for this study was confined to a 40 × 40-km area (160 000 ha; red square in Fig. 1), coinciding with the area depicted in the participatory painting workshop, as well as some of the surrounding areas mentioned during fieldwork discussions in terms of nature conservation, and farmland expansion (i.e. wetland and mountain forest). Details of the methods used to create the LULC data for the three time slices are provided in Leemhuis et al. (2017).

## Results and discussion

### Local perceptions of socio-economic and environmental change

**Fig. 3:**
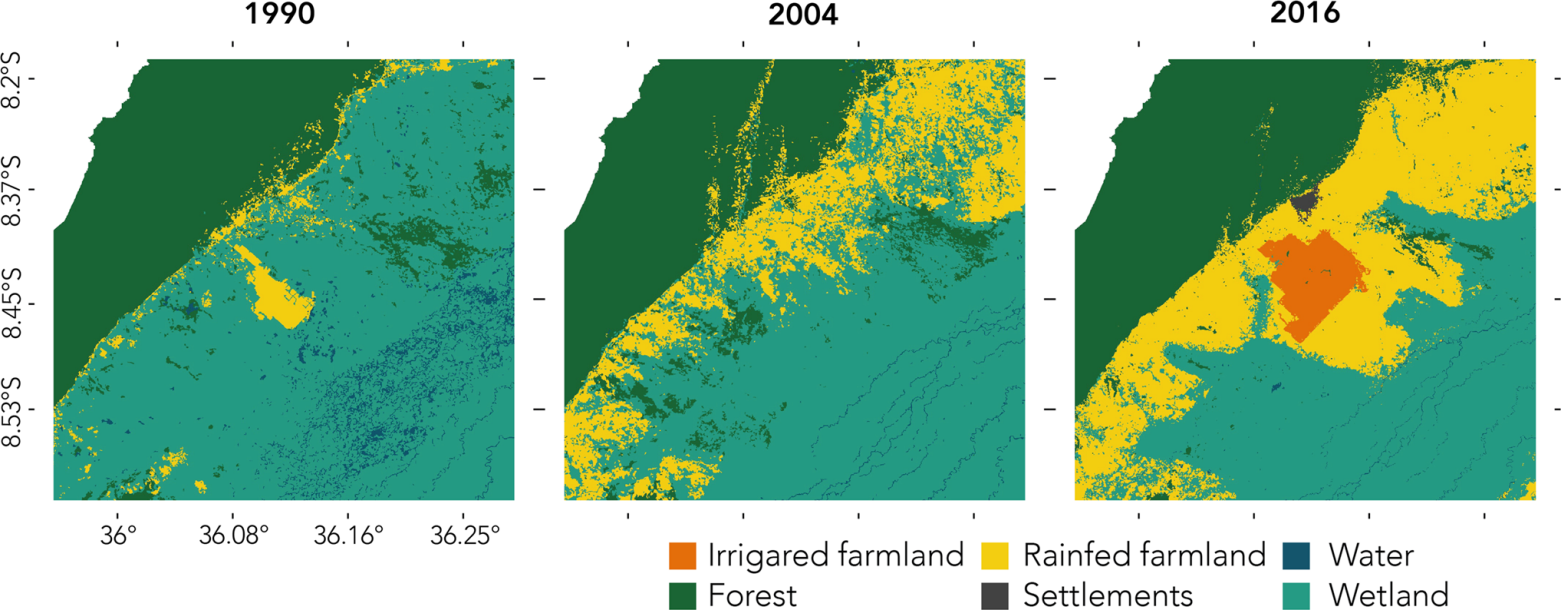
Land cover classes for year 1990, 2004 and 2014. The most visible land-use change is the farmland expansion (yellow) to the wetland area (turquoise). Land acquired by KPL is visible in orange

Local narratives of change highlight increased pressures on land and water resources, which in turn have altered the availability and accessibility of natural resources for local communities. Environmental changes have created new socio-economic challenges since few local residents have been able to find satisfactory alternatives to replace their previous economic activities of farming, fishing and pastoralism. Participants trace these pressures to multiple processes, including rapid population growth, in-migration of people and cattle, nature conservation and establishment of large-scale agriculture. In Johansson and Isgren (2017), social and environmental changes are depicted as paintings, and show, e.g. farmland and settlement expansion, deforestation, wetland degradation, decreased water quantity and quality in rivers and reduced fish and wildlife (Fig. 3).

People explain that the largest driver of environmental change is farmland expansion to the wetland area, both due to the establishment of large-scale rice plantation by KPL and population growth, which has caused a shift in small-scale farming areas and settlements towards the wetlands. Participants also describe that wetlands have been degraded due to rapid increase of pastoralists and cattle to the area over the last decade. These drivers of change have also been identified over the entire Kilombero Valley (Leemhuis et al. 2017; Msofe et al. 2019).

Another significant environmental change mentioned during fieldwork is the reduction of forest cover. Participants describe three different forests in the area: one with large trees for timber that is located far away from the village, towards the wetland, which has not changed much due to its distance from the village. Another forest close to the village where the paintings were made is described to have decreased rapidly due to increased fuelwood collection, described as a consequence of the removal of shrubland areas for the large-scale rice plantation. A third area is the protected mountain forest area, which is described to have been rapidly degraded over the last decade due to illegal activities including farming, charcoal production and collection of timber and fuelwood.

Finally, participants expressed concerns about changing dynamics of water resources, such as a decline in river water. They trace this to irrigation, and further believe that forest degradation and deforestation affects rainfall and contributes to lower water levels in the rivers. Fishermen describe that lower water levels have negatively affected the fish stocks in the rivers, especially in the river where KPL pumps their irrigation water. Fishermen also trace the lower fish stocks to overfishing, mainly as a consequence of increased and intensified fishing activities (e.g. people turn to fishing when they no longer can farm, and loss of swamps where they used to fish that have been drained for establishing large-scale rice plantations).

### Land change detection

**Fig. 4:**
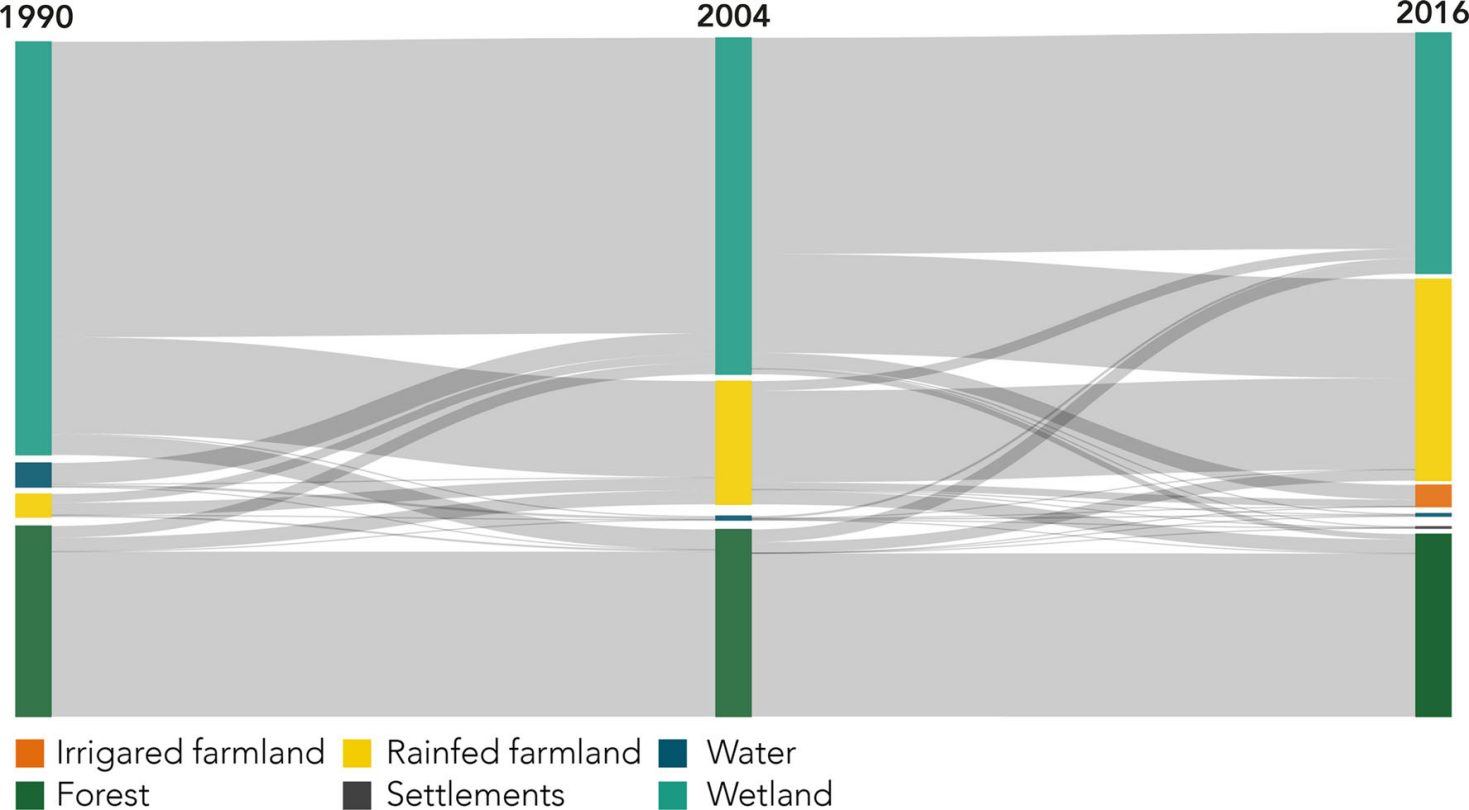
Sankey diagram of the changes in land cover from year 1990, to 2004, and 2016. The diagram shows what land cover has changed to what, as well as how the shares of different land covers have changed over the years

**Fig. 5:**
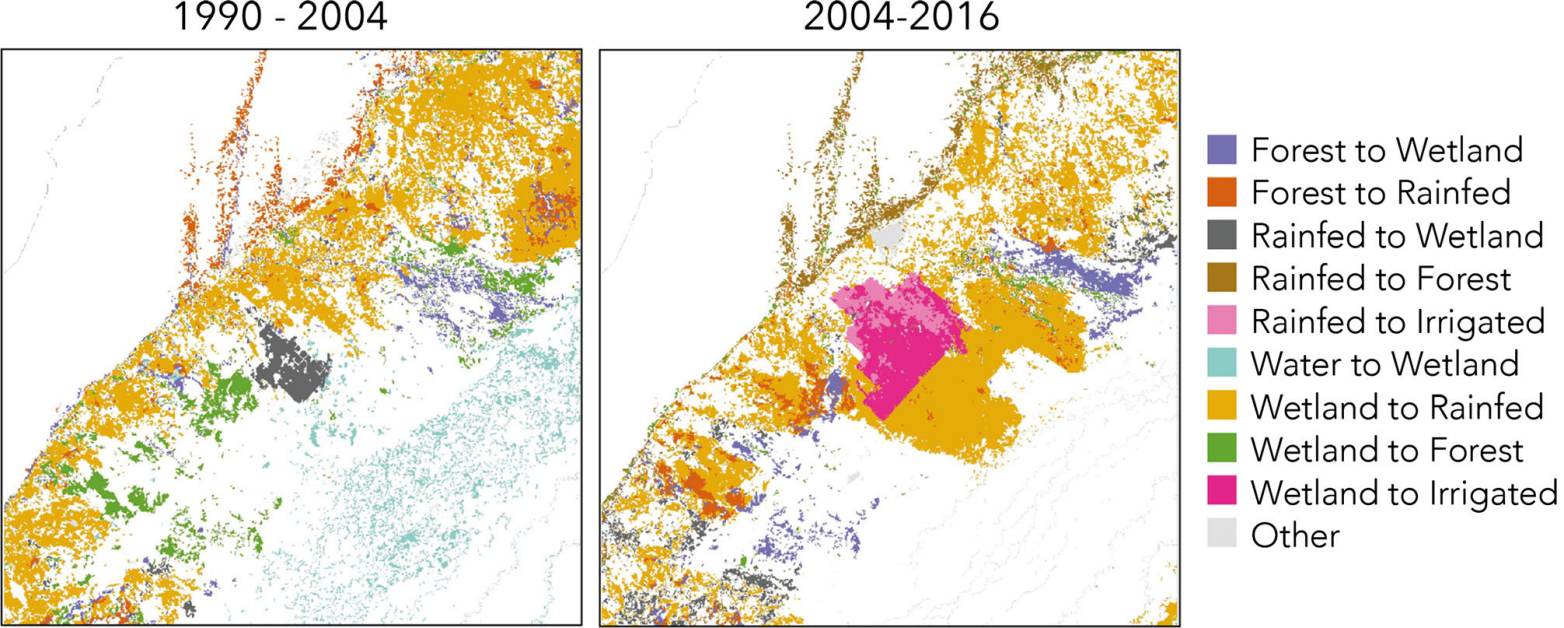
Land change detection of changing pixels between 1990 and 2004, and 2004 and 2016. Pixel categories that represent less than 1% of the image have been grouped as “other”, and coloured in grey. White areas represent pixels or areas that have not changed between the years of comparison

The remote sensing analysis from 1990, 2004 and 2016 shows that the biggest changes are seen in wetland areas (decreased to cover 26% less of the total area), and rainfed farmland (increased by 27%; Table 2). Forests and water bodies have not changed to any large extent, and since 2016, irrigated farmland accounts for 3% of the total LULC of the classified area. Figure 4 shows the share of different LULC for each year, and what they have changed into. LULC changes are also mapped in Fig. 5 in order to see the spatial distribution of change.

**Table 2:** Total area and fraction of land covers for land cover classifications of 1990, 2004 and 2016. Columns to the right also indicate the % change of land covers between the three time periods

	1990		2004		2016		1990–2004	2004–2016	1990–2016
	Area (km^2^)	%	Area (km^2^)	%	Area (km^2^)	%	% change	% change	% change
Wetland	975.1	63	794.4	52	568.6	37	− 12	− 15	− 26
Forest	450.7	29	442.5	29	431.7	28	− 1	− 1	− 1
Rainfed farmland	54.8	4	291.1	19	475.6	31	15	12	27
Water	57.4	4	9.9	1	6.6	0	− 3	0	− 3
Irrigated farmland	0	0	0	0	51.4	3	0	3	3
Settlement	0	0	0	0	3.9	0	0	0	0

Only rainfed agriculture is identified in the 1990 and 2004 images because there was no large-scale irrigation in the study area at the time. Irrigation infrastructure was set up after KPL took over Mgneta Farm in 2008 and is a new land category (West 2014). The classification shows that rainfed agriculture is the land use that has increased the most between 1990 and 2016. Until 2004, 15.4% (236 km^2^) of the pixels changed to rainfed agriculture, and 12% (184 km^2^) until 2016, mainly at the expense of wetland areas (yellow areas in Fig. 5, detailed description in Table 2; Tables S4–S5).

Wetland is the land cover that decreased the most between 1990 and 2016. Until 2004, 11.8% (181 km^2^) of the wetland area was lost, and additionally 14.7% (226 km^2^) until 2016. Most wetland areas have changed to rainfed agriculture (15%), and also to irrigated agriculture (2%). The classification shows minor changes in forest, which decreased by as little as 0.5%, and 3.1% between 1990 to 2004, and 2004 to 2016. Surface water areas have decreased slightly by 50 km^2^ (3.4%) between 1990 and 2016.

### Comparing perceptions on the ground with observations from space

#### Changes in forest cover

**Fig. 6:**
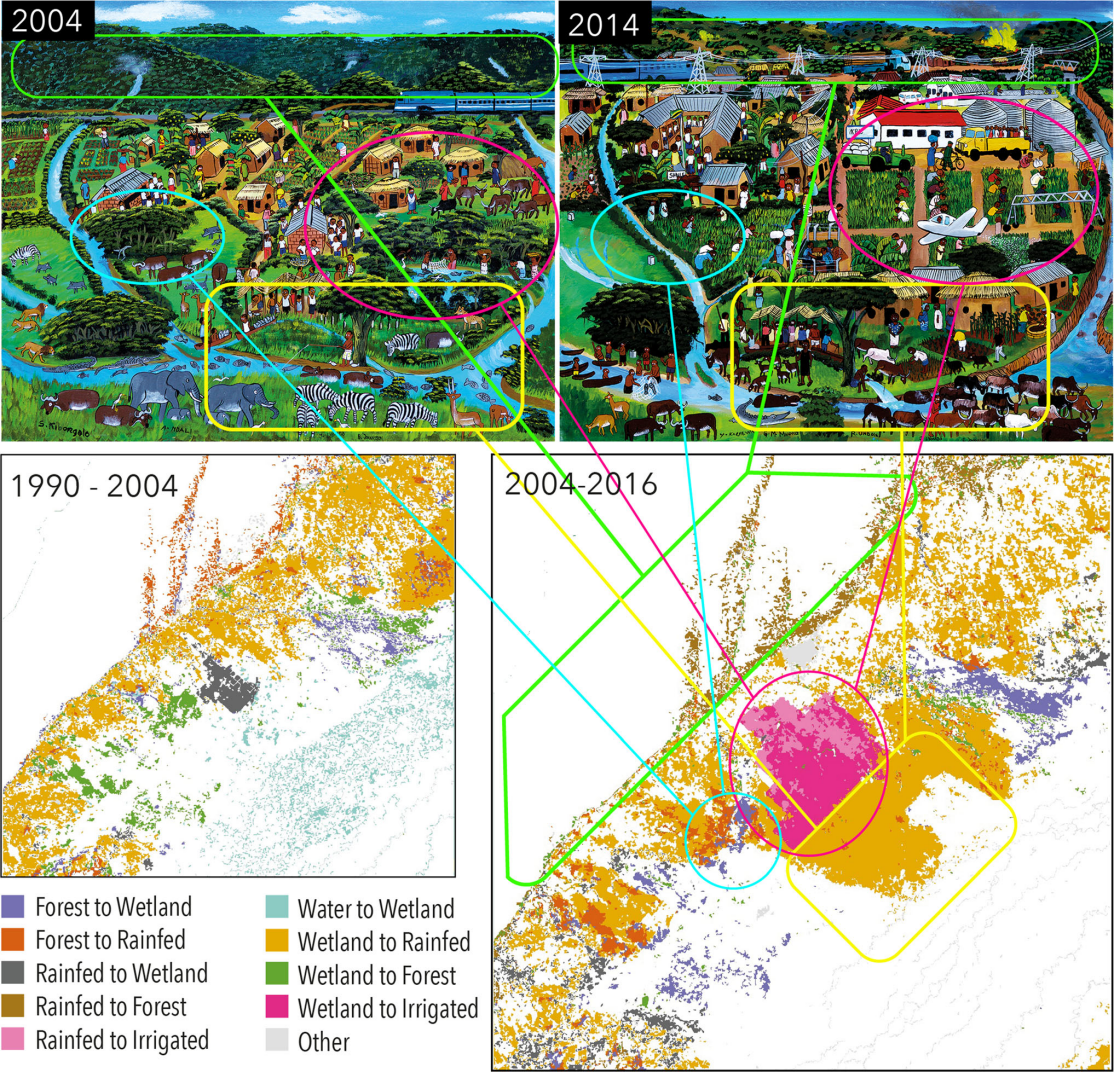
Comparing paintings of the past and the present with land change detection through remote sensing. Narratives and corresponding observations of environmental change are marked and highlighted in different colours. Green circles show the mountain forest as visualised in the paintings, and the corresponding area of the satellite image, mainly showing no change (white) and rainfed agriculture to forest (brown). Blue circles show forest loss in the valley as described in the paintings, and corresponding areas in the land cover classification (forest to rainfed, and forest to wetland). Pink circles show changes from wetland and rainfed farmland to irrigated farmland. Yellow circles show agricultural and pastoral activities expanding over the wetland area, which are seen as wetland to rainfed in the land change detection

Three types of forest areas were mentioned during fieldwork, one that is still intact since it is “far away” in the wetland area, another area close to the settlements that now has disappeared completely and the largest forested area in the mountains that is described as heavily degraded due to illegal deforestation activities. The only deforestation that could be identified in the land change detection was the missing forest areas in the valley (blue circle in Fig. 6). The degradation and deforestation of the mountain forest could not be confirmed by the remote sensing mapping of the case-study area (green circle in Fig. 6). The overarching trend over the entire Kilombero Valley, however, shows a 10% decrease in forest cover between 1990 and 2016 (Leemhuis et al. 2017; Msofe et al. 2019).

A narrative walk with a forest ranger in the mountains was conducted during fieldwork in 2016 in order to verify the local perceptions of deforestation (see transect in Fig. S2). The vast degradation of the mountains was not obvious when viewing the forest from the valley. However, when walking in the forest it was clear that there were numerous cleared plots for farming that were not visible from the outside. This also points to sources of misinterpretation of remotely sensed data, related to issues with spatial resolution, as well as definitions and classification of LULC types. Two plausible explanations for not being able to observe more drastic deforestation trends are that either the deforested areas are smaller than the resolution of the 20-m imagery, or that deforestation occurs in understory clearings with rapid re-growth of grass and shrubs and thus not visible to the satellite since grasslands and shrubland were not part of the classification. Forest degradation for fuelwood and other non-timber forest products is notoriously difficult to observe using a coarse or medium resolution of satellite images, unless the exploitation is intense (DeFries 2008).

#### Changes in farmland and wetland

Farmland expansions and increased pastoral activities in the wetland area have caused conflicts between conservation authorities and local land users (Nindi et al. 2014). The identification of wetland loss due to farmland expansion (both subsistence farming and large-scale rice plantation) was found through both research approaches (yellow and pink circles in Fig. 6). Local participants, however, also attributed part of the wetland degradation to grassland expansion due to an increase of pastoralists and cattle to the area over the last decade, it is therefore likely that farmland and wetland are overestimated in the classification. Changes in grasslands were not investigated through remote sensing, since the lack of historical data and high-resolution imagery made it difficult to discern this LULC class for 1990 and 2004 (thus not providing a solid baseline). Msofe et al. (2019), however, estimate that grasslands in Kilombero Valley have increased by 13% between 1990 and 2016, and agricultural land increased by 11%.

#### Changes in water resources

Local participants express concerns about the changes in water resources in the area. This primarily relates to changes in precipitation patterns, and heavy use of water for irrigation by KPL. The absence of clear trends in water bodies is related to limitations in the geographical extent of the case-study area. However, studies over Kilombero Valley point to minor changes in water bodies between 1990 and 2016 (0.7% decrease). The lack of clear trends is related to the low temporal resolution (only three time stamps), and the high variability and seasonality of water flows. Detailed data on river flow, and irrigation extractions, would be needed to accurately assess changes in water dynamics in the area, but the lack of historical data makes it impossible to compare current water extractions with a baseline. It is, however, reasonable to assume that local hydrology has changed as an effect of the extensive sprinkler irrigation systems implemented by KPL, and other human influences on the hydrologic cycle (Sivapalan et al. 2012).

### Strengths and limitations of the two research approaches

Since mixed methods provide a more nuanced understanding than one method in isolation, the different modes of knowledge production can be used uncover new research gaps (1) where experienced and observed LULC changes diverge, or (2) where perceptions and narratives are part of a problem-feeding process that point to local concerns that need to be further explored (Persson et al. 2018).

The results obtained by mixing top-down and bottom-up research approaches made it possible to see where local perceptions of change align or diverge with measured change. It is in the divergence that it is possible to identify new research gaps, as well as strengths, and weaknesses of the chosen research approaches. Political, historical and economic interests and agendas affect knowledge production, which makes it difficult to find the truth, particularly in areas where there are unequal power relations between different natural resource users. In this case, we removed some of these subjectivities with mixed research approaches.

For example, the contrasting results regarding forest change shows that all knowledge is partial, contextual and linked to how it is created (Nightingale 2016). People who actively engage with natural resources have a sensitivity to register critical and unusual signs and signals in the environment, and can indicate which LULC classes are important, where they are located and how they change (Berkes 2010). Without merging results from the two research approaches, it would not have been clear that there is a disagreement in current narratives of forest cover. It is in the disagreement that uncertainties of different research approaches can be identified, and where it is important to integrate qualitative and quantitative approaches for a better description of socio-environmental change. The mismatch between experienced change and identified change raises a warning flag for decision makers who base policies solely on quantitative estimates of LULC change, or problem formulations based on one method in isolation.

Participants discussed many social effects linked to land-use changes. One such example is that KPL spread fertilisers on their rice fields with a small airplane. People that passed by the painting workshop further explained that the spread of chemicals by air has damaged small-scale rice fields west of the plantation due to wind directions and too high concentrations of chemicals (despite buffer zones around company plantations). Another common effect linked to the spread of chemicals by air is that wells close to the company plantations become polluted after a flyover, and people get itchy skin. These types of experiences (negative effects on harvests, and health) can be used in a problem-feeding process to identify research gaps and new research questions based on local needs and concerns (Persson et al. 2018). As foreign investments in agriculture play an increasingly important role in raising pressures on land and water resources, such local experiences also highlight the urgency to develop cross-scale policies for locally inclusive development that also incorporates the Sustainable Development Goals (Griggs et al. 2013).

Our approach posits that policies must be established and strengthened at multiple scales to facilitate a transformation towards sustainable pathways for agricultural development. These include policies in global trade agreements, importing regions and countries, as well as countries where land is acquired (Dong et al. 2019). In order to break the power asymmetries between agribusinesses and local farmers, governments of importing, and targeted countries, should demand that agribusinesses follow guidelines for responsible investments like those developed by the Committee on World Food Security (2014). District-level authorities in areas of land acquisitions need to apply more inclusive models for deciding what is required by foreign investors, for example, what crops are most suitable for the local context, or what jobs and education opportunities, are needed.

## Conclusion

This study explores how distant actors affect people and nature elsewhere, by investigating landscape changes in an area that experience large-scale land acquisitions in Kilombero Valley, Tanzania. We use already established LULC classifications, and narratives from participatory research to study drivers and impacts of environmental change.

Remote sensing and participatory research approaches are two complementary methods for investigating socio-economic and environmental change, in particular, in areas where there is a lack of historical data (e.g. maps, photos, satellite images with high temporal and spatial resolution and other environmental data), which makes it difficult to compare the current state with a baseline of the past. Remote sensing is strong in its ability to quantify and map patterns of change, but provides limited descriptions of the underlying drivers and experiences of change. Participatory methods, however, add detail regarding the underlying socio-economic drivers and lived experiences of environmental change. Mixing methods is therefore particularly important when investigating and validating local experiences of change in areas of natural resource conflicts, such as large-scale land acquisitions. Therefore, we highlight the importance to integrate lived experiences of change into natural resource management, and not base land-use decisions on quantitative estimates in isolation. Drawing on local knowledge and perceptions is crucial for making informed decisions based on local challenges and concerns, and for co-developing pathways towards a sustainable future for all.

## Electronic supplementary material

Below is the link to the electronic supplementary material.

Supplementary material 1 (PDF 5793 kb)
