# Supplementary material for: Mapping and quantifying perceptions of environmental change in Kilombero Valley, Tanzania
Source: Ambio. 2019 Jul 25;49(2):557–68. doi: 10.1007/s13280-019-01226-6 (PMC6965049; doi:10.1007/s13280-019-01226-6)
Supplement: Supplementary file 1 — Supplementary material 1 (PDF 5793 kb) [file 13280_2019_1226_MOESM1_ESM.pdf]

Ambio

Electronic Supplementary Material
This supplementary material has not been peer reviewed.

Title: Mapping and Quantifying Perceptions of Environmental Change in Kilombero Valley, Tanzania.

Authors: Emma Li Johansson, Abdulhakim Mohamed Abdi

Table S1. Land acquisitions in Kilombero Valley, investor name, investor countries, contract size, and purpose of production according to the Land Matrix database (2019).

Company	Investor countries	Size (ha)	Production
Kilombero Farms Company Ltd.	Tanzania (100%, founded by Tanzanian and Canadian farmers)	405	Rice
Kilombero Plantations Ltd.	UK (92%), Tanzania (8%)	5 818	Rice
Green Resources Ltd	Norway (100%)	92 500	Eucalyptus, Pine, Teak
New Forests Company Tanzania UK Ltd.	UK (87%), Tanzania (13%)	8 098	Eucalyptus, Pine
Kilombero Sugar Company Ltd. (Illovo)	South Africa (55%), UK (20%), Tanzania (25%)	6 774	Sugarcane
Udzungwa Forest Project (UFP)	Tanzania	Unknown	Conservation
KCY Mpanga Co Ltd. (Kilimo cha Yesu)	Switzerland	263	Rice
Kilombero Valley Teak Company	Finland and the US	28 132	Teak

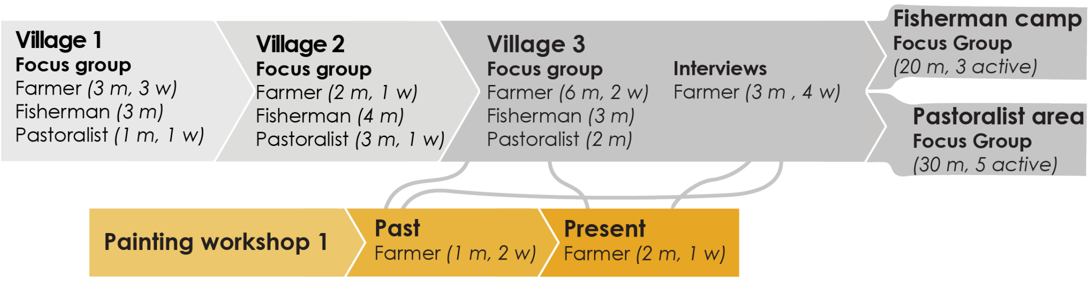

Figure S1. Gender, age, and livelihood representation of people participating in focus group discussions, interviews, and painting workshops. Adapted from Johansson & Isgren (2017).

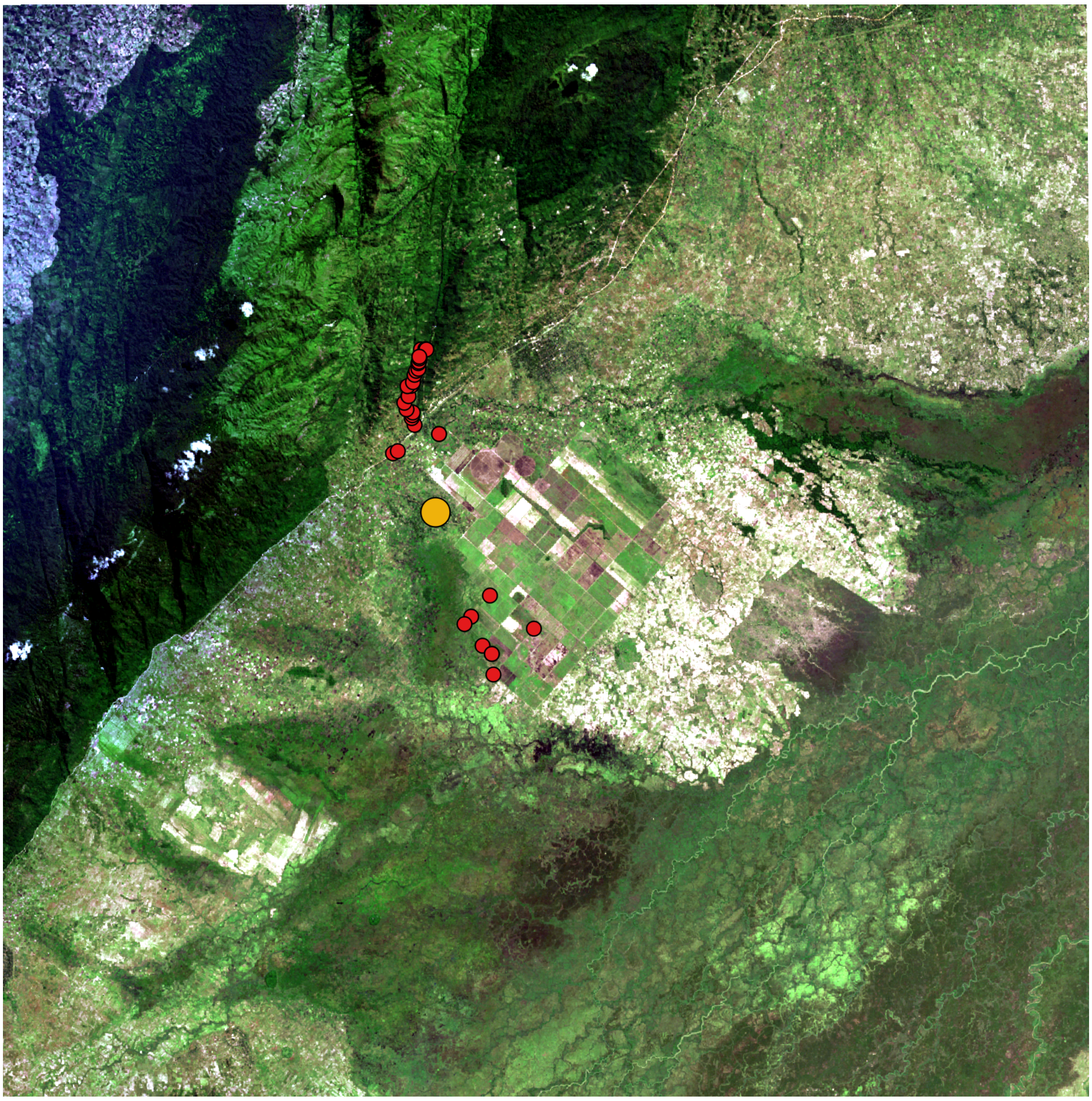

Figure S2. GPS point of field observations during a transect walk in the mountain forest (red points north of yellow point), and along the large-scale rice plantation (red points south of yellow point). The yellow point indicates the centre of Mkangawalo village.

Accuracy assessment of land cover classification

Accuracy measures show to what extent the map user and producer can trust the classification for each individual class. The user's accuracy is calculated by dividing the number of correctly classified pixels in each category by the number of pixels that were classified as that category. The measure shows false positives of the classification, which are cases where pixels of a certain class are over-represented and should have been classified as something else according to ground truth data. For example when ground truth data identifies a pixel as farmland, but was classified as wetland. Producer's accuracy on the contrary shows the false negatives, which are pixels that are under-represented in the classification. For example when the classified image identifies a pixel as forest that is farmland according to ground truth data. The producer's accuracy is

calculated by dividing the correctly classified pixels in each category by the number of ground truth points for that category.

Table S2. ORIGINAL VALUES OF VALIDATION OF LAND COVER CLASSIFICATION BY Leemhuis et al. (2017).

**1990**					
**Land_Cover_Use**	**Forest**	**Arable_land**	**Wetland**	**Water**	**Total**
**Forest**	93	5	11	2	111
**Arable_land**	2	77	12	0	91
**Wetland**	5	15	80	13	113
**Water**	0	1	0	84	85
**Total**	100	98	103	99	400

**2004**					
**Land_Cover_Use**	**Forest**	**Arable_land**	**Wetland**	**Water**	**Total**
**Forest**	94	4	2	0	100
**Arable_land**	10	76	14	0	100
**Wetland**	0	8	92	0	100
**Water**	2	0	12	86	100
**Total**	106	88	120	86	

**2016**					
**Land_Cover_Use**	**Forest**	**Arable_land**	**Wetland**	**Water**	**Total**
**Forest**	92	8	0	0	100
**Arable_land**	0	96	4	0	100
**Wetland**	2	18	80	0	100
**Water**	4	2	14	80	100
**Total**	98	124	98	80	

Table S3. ADJUSTED VALIDATION VALUES (ACCORDING TO Olofsson et al. (2014)) FOR LAND COVER CLASSIFICATION BY Leemhuis et al. (2017).

Land_Cover_Use 1990	Forest	Arable_land	Wetland	Water	Total
**Forest**	0,5712	0,0307	0,0676	0,0123	0,6818
**Arable_land**	0,0009	0,0338	0,0053	0,0000	0,0399
**Wetland**	0,0117	0,0352	0,1878	0,0305	0,2653
**Water**	0,0000	0,0002	0,0000	0,0129	0,0131
**Total**	0,5838	0,0998	0,2606	0,0557	
**Overall accuracy**					**80,57%**
**Users accuracy**	83,78%	84,62%	70,80%	98,82%	**84,50%**
**Producers accuracy**	97,84%	33,81%	72,06%	23,18%	**56,72%**

Land Cover Use 2004	Forest	Arable_land	Wetland	Water	Total
**Forest**	0,6813	0,0290	0,0145	0,0000	0,7248
**Arable_land**	0,0103	0,0782	0,0144	0,0000	0,1029
**Wetland**	0,0000	0,0133	0,1533	0,0000	0,1666
**Water**	0,0001	0,0000	0,0007	0,0049	0,0057
**Total**	0,6917	0,1205	0,1829	0,0049	1,0000
**Overall accuracy**					**91,77%**
**Users accuracy**	94,00%	76,00%	92,00%	86,00%	**87,00%**
**Producers accuracy**	98,50%	64,89%	83,82%	100,00%	**86,80%**

Land_Cover_Use 2016	Forest	Arable_land	Wetland	Water	Total
**Forest**	0,6168	0,0536	0,0000	0,0000	0,6705
**Arable_land**	0,0000	0,1983	0,0083	0,0000	0,2066
**Wetland**	0,0024	0,0214	0,0950	0,0000	0,1187
**Water**	0,0002	0,0001	0,0006	0,0034	0,0042
**Total**	0,6194	0,2734	0,1038	0,0034	1,0000
**Overall accuracy**					**91,35%**
**Users accuracy**	92,00%	96,00%	80,00%	80,00%	**87,00%**
**Producers accuracy**	99,59%	72,53%	91,47%	100,00%	**90,90%**

Table S4. Cross-tabulation of land cover changes between 1990 and 2004.

	Forest	Rainfed	Water	Wetland	**Total 1990**
Forest	25.3%	2%	0.1%	2%	**29%**
Rainfed	0.3%	1.9%	0.003%	1%	**4%**
Water	0.03%	0%	0.4%	3%	**4%**
Wetland	3%	15%	0%	45.3%	**63%**
**Total 2004**	**29%**	**19%**	**1%**	**52%**	

Table S5. Cross-tabulation of land cover changes between 2004 and 2016.

	Forest	Irrigated	Rainfed	Settlements	Water	Wetland	**Total 2004**
Forest	25%	0.01%	2%	0.1%	0.01%	2%	**29%**
Irrigated	0%	0%	0%	0%	0%	0%	**0%**
Rainfed	2%	1%	14%	0.2%	0.01%	2%	**19%**
Settlements	0%	0%	0%	0%	0%	0%	**0%**
Water	0.1%	0.0%	0.01%	0.0%	0.2%	0.4%	**1%**
Wetland	1%	2%	15%	0.02%	0.2%	33%	**52%**
**Total 2016**	**28%**	**3%**	**31%**	**0%**	**0%**	**37%**	

Census data and estimations of farmland expansion due to population growth

Population data is an additional source used to estimate the potential farmland expansion due to population growth in the area. The population and socio-economic data is based on census data from 2002 and 2012 (NBS, 2012), but also from region-level documents (NBS, 2016), and village-level land use plans for the three villages obtained from district level authorities. The land-use plans are written in Swahili and were mainly used to obtain information about village boundaries, land use and land cover, and detailed population data for the three villages that lease land to the foreign agribusiness.

We use statistics from NBS (2016) to estimate total farmland increase due to population growth in the study area between 2002 and 2012. We base the estimates on information that 78.7 % of the rural population in Kilombero District are farmers; that a household consists of an average 4.4 people; and that the average farm size of a household is 2.2 ha.

Based on the information given in the different census documents (see materials and methods), the population of Kilombero District increased by 21% between 2002 and 2012. The district consists of 18 wards, of which the three villages included in this study are part of Mchombe Ward, which had a population increase of 30% (27,207 to 38,651)

between 2002 and 2012. In absolute numbers, it is the ward that has had the biggest population growth in Kilombero District (11,444 people; Population and Housing Census (2012)). The villages Lukolongo, Mkangawalo, Mngeta (grey areas in Figure 1) had a total population of 23,000 in 2012 (Village-level land use plans, 2012).

We estimate that the total farmland increase due to population growth in the entire Kilombero District corresponds to 33,946 ha. Based on the share of population change for the different wards, we estimate that the farmland expansion due to population growth between 2002 and 2012 in Mchombe ward corresponds to about 4,500 ha.

Table S6. Land Acquisitions in Production in Tanzania as reported by Land Matrix (2017).

Deal ID	Location	Investor name	Investor country	Contract size (ha)	Production size (ha)	Crop	Intention	Implementation status	Nature of the deal
**1909**	Bukoba, Tanzania	Super Group of Companies Ltd.	Tanzania	17000	9000	Sugar Cane	Biofuels, Food crops, Renewable Energy	[2001] In operation (production)	Lease / Concession
**3889**	Moshi, Tanzania	Alteo Ltd, Government of Tanzania	Mauritius, Tanzania	15800	8000	Sugar Cane	Food crops, Renewable Energy	In operation (production)	NA
**3021**	Kilombero, Tanzania, Ulanga, Tanzania	Global Environment Fund, Finnish Fund for Development Cooperation (Finnfund)	USA, Finland	28132	4748	Teak	For carbon sequestration/REDD, For wood and fibre	[2011] In operation (production)	Exploitation license
**1876**	Iringa, Tanzania, Dabaga, Tanzania, Idete, Tanzania	New Forests Company Holdings, Government of Tanzania	UK, Tanzania	8098	4699	Eucalyptus, Pine, Trees	For carbon sequestration/REDD, For wood and fibre, Other (please specify)	In operation (production)	Outright Purchase, Exploitation license
**1839**	Mngeta, Kilombero, Tanzania	Agrica, Rufiji Basin Development Authority (RUBADA)	UK, Tanzania	5818	4178	Rice	Food crops	[2010] In operation (production)	Outright Purchase
**5192**	Arusha, Tanzania	Luke Edwards, Tanzania Breweries Limited (TBL)	Tanzania	3845	3845	Barley, Wheat	Food crops, Livestock	In operation (production)	Lease / Concession
**1889**	Mbarali River, Tanzania	ETG Farming, Verus Group	Tanzania, South Africa	7370	3000	Barley, Jatropha, Rice	Biofuels, Food crops	[2012] In operation (production)	Lease / Concession
**1846**	Kisarawe, Tanzania	Thirty Degrees East	Mauritius	8211	2023	Jatropha	Biofuels	Project abandoned	Lease / Concession
**4717**	Pangani, Tanzania, Handeni, Tanzania, Mkinga, Tanzania	Green Resources AS	Norway	10000	1340	Eucalyptus, Pine, Teak, Trees	For carbon sequestration/REDD, For wood and fibre	[2012] Project abandoned	Lease / Concession
**4757**	Ruvuma, Tanzania	Olam International Ltd.	Singapore	2000	1025	Coffee Plant	Conservation, Food crops	[2014] In operation (production)	Lease / Concession
**4281**	Tanga, Tanzania	AgDevCo	UK	2500	1000	Banana, Vegetables	Food crops	[2012] In operation (production)	NA
**1948**	Tanga, Tanzania	K.I. Samen B.V., Holland Dairies, Tanga Dairies Cooperative Union, Katani Ltd.	Netherlands, Tanzania	1000	1000	NA	Livestock	[2012] In operation (production)	Lease / Concession

**1875**	Rufiji River, Tanzania	African Green Oils	Norway	860	360	Oil Palm	Agriunspecified	[2011] Project abandoned	Lease / Concession
**2398**	Rufiji River, Tanzania	Safe Production Ltd	Turkey	5000	300	Corn (Maize), Rice	Food crops	[2007] Project abandoned	NA
**1895**	Bagamoyo, Tanzania	EcoEnergy Africa AB, Government of Tanzania	Sweden, Tanzania	22300	200	Sorghum, Sugar Cane	Biofuels, Food crops, For wood and fibre, Other (please specify), Renewable Energy	[2008] Startup phase (no production)	Lease / Concession
**1880**	Dodoma, Tanzania, Handeni, Tanzania, Kongwa, Tanzania	Donesta Ltd & Savannah Biofuels LTD	Tanzania	7000	200	Jatropha, Sun Flower	Biofuels	[2012] Project abandoned	NA
**1872**	Kigoma, Tanzania	FELISA, Unnamed investor 261	Belgium, Tanzania	4258	100	Sun Flower, Corn (Maize)	Food crops	[2014] In operation (production)	Outright Purchase
**1866**	Nainokwe, Tanzania	Bioshape Holding	Netherlands	34000	70	Jatropha, Teak	Biofuels	[2010] Project abandoned	Lease / Concession
**4925**	Wami River, Tanzania	Obtala Resources Ltd , Unknown	UK, Tanzania	1200	30	Mango, Banana, Fruit, Vegetables	Food crops	[2015] In operation (production)	Lease / Concession
**1854**	Ikwiriri, Tanzania	VitaGrain, Rufiji Basin Development Authority (RUBADA)	Singapore, Tanzania	13000	2	Sorghum, Corn (Maize), Rice (hybrid), Soya Beans	Food crops	In operation (production)	Lease / Concession

LITERATURE CITED

BREIMAN, L. 2001. Random Forests. *Machine Learning,* 45**,** 5-32.

JOHANSSON, E. L. & ISGREN, E. 2017. Local perceptions of land-use change: using participatory art to reveal direct and indirect socioenvironmental effects of land acquisitions in Kilombero Valley, Tanzania. *Ecology and Society,* 22.

LEEMHUIS, C., THONFELD, F., NÄSCHEN, K., STEINBACH, S., MURO, J., STRAUCH, A., LÓPEZ, A., DACONTO, G., GAMES, I. & DIEKKRÜGER, B. 2017. Sustainability in the food-water-ecosystem nexus: the role of land use and land cover change for water resources and ecosystems in the Kilombero Wetland, Tanzania. *Sustainability,* 9**,** 1513.

OLOFSSON, P., FOODY, G. M., HEROLD, M., STEHMAN, S. V., WOODCOCK, C. E. & WULDER, M. A. 2014. Good practices for estimating area and assessing accuracy of land change. *Remote Sensing of Environment,* 148**,** 42-57.
